# Integrating artificial intelligence in drug discovery and early drug development: a transformative approach

**DOI:** 10.1186/s40364-025-00758-2

**Published:** 2025-03-14

**Authors:** Alberto Ocana, Atanasio Pandiella, Cristian Privat, Iván Bravo, Miguel Luengo-Oroz, Eitan Amir, Balazs Gyorffy

**Affiliations:** 1 Experimental Therapeutics in Cancer Unit, Medical Oncology Department, Instituto de Investigación Sanitaria San Carlos (IdISSC), Hospital Clínico San Carlos and CIBERONC, Madrid, Spain; 2 INTHEOS-CEU-START Catedra, Facultad de Medicina, Universidad CEU San Pablo, 28668 Boadilla del Monte, Madrid, Spain; 3 Instituto de Biología Molecular y Celular del Cáncer, CSIC, IBSAL and CIBERONC, Salamanca, 37007 Spain; 4 CancerAppy, Av Ribera de Axpe, 28, Erando, 48950 Vizcaya Spain; 5 Facultad de Farmacia, Universidad de Castilla La Mancha, Albacete, Spain; 6 Spotlab, Paseo Juan XXIII 36b, Albacete, 28007 Spain; 7 Princess Margaret Cancer Center, Toronto, Canada; 8 Department of Bioinformatics, Semmelweis University, Tűzoltó U. 7-9, Budapest, 1094 Hungary; 9 Research Centre for Natural Sciences, Hungarian Research Network, Magyar Tudosok Korutja 2, Budapest, 1117 Hungary; 10 Department of Biophysics, Medical School, University of Pecs, Pecs, 7624 Hungary

**Keywords:** Artificial intelligence, Drug discovery, Target identification, Early clinical development, Clinical trials, Neural networks, Deep learning, Oncology, AlphaFold

## Abstract

Artificial intelligence (AI) can transform drug discovery and early drug development by addressing inefficiencies in traditional methods, which often face high costs, long timelines, and low success rates. In this review we provide an overview of how to integrate AI to the current drug discovery and development process, as it can enhance activities like target identification, drug discovery, and early clinical development. Through multiomics data analysis and network-based approaches, AI can help to identify novel oncogenic vulnerabilities and key therapeutic targets. AI models, such as AlphaFold, predict protein structures with high accuracy, aiding druggability assessments and structure-based drug design. AI also facilitates virtual screening and de novo drug design, creating optimized molecular structures for specific biological properties. In early clinical development, AI supports patient recruitment by analyzing electronic health records and improves trial design through predictive modeling, protocol optimization, and adaptive strategies. Innovations like synthetic control arms and digital twins can reduce logistical and ethical challenges by simulating outcomes using real-world or virtual patient data. Despite these advancements, limitations remain. AI models may be biased if trained on unrepresentative datasets, and reliance on historical or synthetic data can lead to overfitting or lack generalizability. Ethical and regulatory issues, such as data privacy, also challenge the implementation of AI. In conclusion, in this review we provide a comprehensive overview about how to integrate AI into current processes. These efforts, although they will demand collaboration between professionals, and robust data quality, have a transformative potential to accelerate drug development.

## Highlights

Artificial intelligence can transform drug discovery and early drug development.

AI can facilitate target identification with multiomics data and network-based approaches.

AlphaFold can predict protein structures with high accuracy/druggability assessments.

AI can help in structure/ligand-based drug design, de novo design and lead optimization pb and lead optimization processes.

In trial design AI can boost predictive and adaptive modelling, synthetic arms or digital twins.

## Background

While the traditional drug discovery model has yielded numerous life-saving treatments, it faces challenges which result in delaying the availability of new cancer therapies for patients. For instance, on average, bringing a new drug to market takes 10–15 years and costs approximately $2.6 billion considering both direct and indirect costs [1, 2]. Additionally, the failure rate is extremely high, with less than 10% of drug candidates reaching the market successfully [2]. Taking into consideration these numbers, there is an unmet need to identify ways to reduce and optimize the entire drug development process. Indeed, most failures in clinical development are associated with safety and lack of activity, indicating the need for an improvement in preclinical processes like target selection/identification and drug discovery [3]. Artificial intelligence (AI) has gained momentum in the biomedical field during the last few years as it permits the analysis and interpretation of a large amount of data that cannot be performed with traditional statistical methods [4].

While traditional modalities in drug discovery and early clinical development have proven to be effective, as evidenced by the successful development and approval of novel agents [5], there remains a significant opportunity to optimize these models using AI. Furthermore, AI presents the potential to introduce innovative approaches that can transform and accelerate these processes.

In this review, we focus on strategies to incorporate AI in all the processes associated with early clinical development in cancer research, starting from the evaluation of the classical modalities. Our aim is to provide a comprehensive overview of these initiatives to improve the drug discovery model.

## Classical modalities in drug discovery and early clinical development

**Fig. 1:**
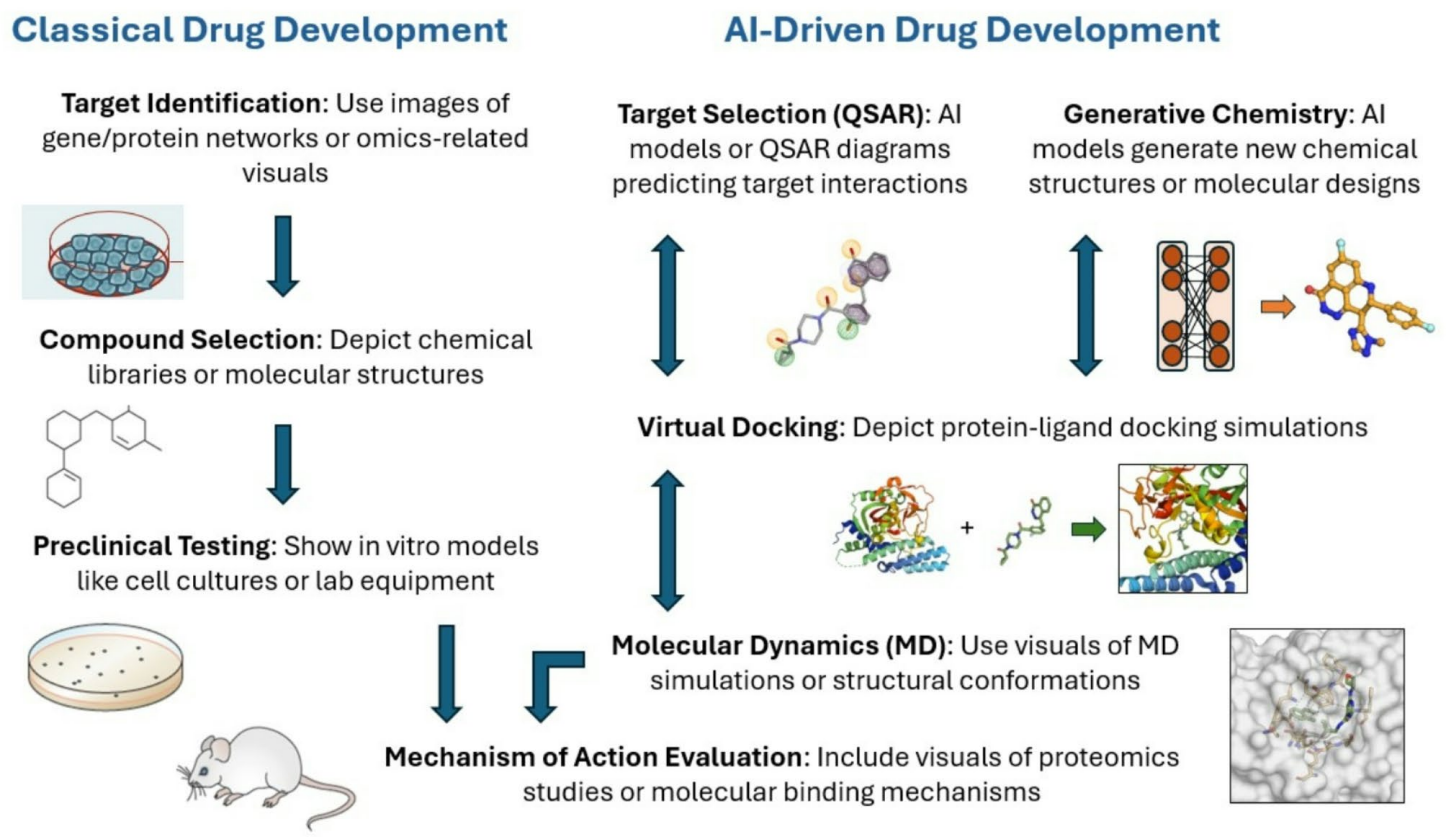
Description of classical versus AI driven drug development

Typically, traditional cancer drug discovery involves a series of processes that begin either with the identification of a therapeutic target or with the selection of a chemical entity with antiproliferative activity [5]. In the latter scenario, experiments involved in the preclinical characterization are long-lasting, expensive, and time-consuming, as there is a need to identify the specific target to which the chemical compound binds, along with additional analysis to characterize the mechanism of action, and potential combination strategies [6]. With the implementation of “omic” techniques including sequencing analysis, novel druggable targets can be identified [7]. In this context, the discovery of chemical entities that would specifically inhibit those proteins is highly desirable. This method using a “personalized model” in which a genomic profile guided the discovery of specific inhibitors and the subsequent selection of patients for that compound, produced more clinical benefit than the classical empiric treatment approach where therapies were given to all patients [8]. Figure 1 represents some of the key steps in the drug discovery process.

### Target identification and drug discovery

Whole genomic analysis reinforced with functional studies has helped in the identification of novel oncogenic vulnerabilities [7, 9, 10]. Experimental methods, including gene knockout studies and high-throughput screening (HTS) like those with CRISPR-Cas9 have been instrumental in elucidating potential targets [11–13]. ​ Some target screening approaches have been conducted under specific conditions where tumors were particularly dependent on a specific genetic alteration or were influenced by changes in the tumor microenvironment [10, 14–16]. These methods have been crucial to identify vulnerabilities such as synthetic lethality interactions [17]. A recent example is the strong genomic dependency between MTAP (methylthioadenosine phosphorylase) deletion and PRMT5 inhibition in various cancers [18, 19]. However, not all proteins are druggable. For a protein to be druggable it needs to exhibit specific characteristics that make it an appropriate target for therapeutic interventions, either through small molecules or biologics, such as antibodies [20]. These characteristics include a well-defined binding site or “pocket” where small molecules can physically bind [20, 21]. This binding site should be accessible and specific enough for a drug to interact with high affinity and modulate the protein’s activity without affecting other proteins. In line with this, the protein must be stable enough to maintain a suitable conformation for a drug to bind effectively. Those proteins that may not maintain a stable structure or lack a drug-binding pocket, are therefore considered as undruggable [22]. Some proteins involved in large protein-protein interactions, have large, flat surfaces that do not easily accommodate small molecules, and others can have highly flexible or dynamic structures which makes binding difficult [23]. Examples of this kind of proteins include transcription factors or scaffolding proteins that regulate multiple cellular processes but lack accessible drug-binding sites [24].

In the discovery of new chemical entities, classical drug discovery relies heavily on HTS techniques to test large libraries of chemical compounds against the identified target [15, 25]. These methods were often paired with structure-activity relationship (SAR) studies, where the biological activity of compounds correlated with their chemical structure to improve efficacy [26]​​. Following these processes, the selected compounds can be modified chemically to enhance their properties, such as potency, selectivity, and pharmacokinetics, while minimizing toxicity and off-target effects. This process is called lead optimization [5].

### Early trial designs

Phase I trials focus primarily on evaluating the safety and optimal dosing of a new drug. The goal is to identify the maximum tolerated dose (MTD) and observe any dose-limiting toxicities (DLTs). Phase I trials are often conducted with small groups of patients, typically involving individuals with advanced cancer who have exhausted standard treatments [27]. Classical designs include the 3 + 3 escalation design in which three patients are enrolled at a starting dose. If no DLTs are observed, three additional patients are treated at a higher dose, but if one patient experiences a DLT, three more patients are treated at the same dose level. Dose escalation continues until DLTs are observed in at least two of six patients, at which point the MTD is determined [28]. Modified 3 + 3 designs or accelerated titration designs can help in the escalation process reducing the number of patients treated at untherapeutic dose levels [29]. These classical methods have limitations as they are time consuming, they do not take in consideration patient heterogeneity and the dose selected is only based on the exposure and safety profile of a very limited number of patients in a short period of time. Additionally, with DLTs focused on acute toxicity, little data are collected on long term safety and tolerability. As such, the information provided to design the next stage of clinical development can be limited.

## Challenges and limitations of classical modalities

There are several limitations, specific to drug discovery and development in cancer, that can be summarized in the following concepts: (1) High Costs and Long Timelines: 10–15 years for a drug candidate to receive regulatory approval [30]; (2) Low Success Rates: approximately 90% of candidates that enter early clinical trials do not reach the market [31]; and (3) Complex Disease Biology: cancer involves complex, interconnected biological pathways that are difficult to target effectively with classical methods​. This complexity often leads to drug development failures when classical models focus on singular targets or simplified pathways [32]​. In these settings, drug combinations are usually needed [33]; (4) Target Druggability: classical approaches are limited in their ability to target complex molecules like protein-protein interactions or “undruggable” targets that lack well-defined binding sites [34]. Some of these undruggable targets are typical in the genesis of cancer.

As the main reasons for failures in drug development are insufficient efficacy and safety levels, methods based on AI could help mitigate challenges in the analysis of multiomics data by improving target identification and predicting druggability, which enhances the overall drug discovery process.

We observed that classical statistical methods were often limited by their reliance on pre-specified parametric models and assumptions that did not hold true for the complex, high-dimensional nature of modern biological datasets. These conventional techniques frequently fell short when tasked with capturing non-linear interactions and managing the heterogeneity inherent in multiomics data [35], necessitating extensive preprocessing and dimensionality reduction. In contrast, AI approaches, particularly those based on deep learning (DL) and ensemble strategies, demonstrated an exceptional ability to autonomously extract meaningful features from noisy and large-scale datasets. By bypassing strict parametric constraints, AI models could integrate diverse data types and uncover subtle, non-linear relationships that were otherwise missed [4]. This paradigm shift not only improves predictive accuracy but also streamlines the data processing pipeline, thereby accelerating the drug discovery and early clinical development process.

Moreover, AI may play a crucial role in optimizing safety by refining lead optimization and streamlining early clinical development, ensuring that drug candidates with greater efficacy and lower toxicity progress to later-stage trials.

In the following section, we will discuss how AI can be applied to improve these critical aspects of drug development.

## Artificial intelligence applied to cancer research

The history of AI applied to cancer research spans several decades, beginning with early computational models and evolving into sophisticated machine learning (ML) algorithms capable of analyzing massive datasets [36, 37]. This evolution reflects the broader progress in AI, with notable advances in DL, neural networks, and data-driven methodologies. In this section, we describe how AI can help to modify some of the mentioned processes (Fig. 1).

### AI in target identification

Target identification is a key step in drug development [5]. AI has become a crucial tool offering the ability to analyze vast datasets, uncover hidden patterns, and propose novel therapeutic targets that may have been overlooked by traditional methods [34, 38]. AI-driven approaches not only streamline the target discovery process but also provide insights into complex biological networks that are key components of the oncogenic processes.

#### AI for large-scale data analysis and network-based target identification

One of the key advantages of AI in target identification is its capacity to analyze large and complex datasets, such as those generated from multiomics studies [39]. AI models, particularly ML and DL algorithms, can process genomic, proteomic, and transcriptomic data to identify key biomarkers and druggable targets [39, 40]​. Often these datasets are too large and complex for traditional analytical methods to handle effectively. By leveraging AI, researchers can integrate different types of biological data, identify relevant patterns, and prioritize potential targets based on their biological significance and druggability [39, 41].

As mentioned previously, most of the biological processes in cancer are not governed by a single gene or protein. Instead, they involve intricate networks of interacting genes, proteins, and pathways. AI excels in network-based approaches by analyzing these biological networks and identifying key nodes (targets) that are critical to disease progression​ [38]​. DL models such as convolutional neural networks (CNNs) and recurrent neural networks (RNNs) can be trained on knowing drug-target interactions and used to predict new ones [41]. Generative adversarial networks (GANs) can help design new molecules that are optimized to bind to specific network nodes, offering an AI-driven approach to drug design [42]. Reinforcement learning (RL) models excel at generating novel molecules with distributions different from the training data sets, making it possible to explore unknown chemical spaces and optimize for specific properties. In addition, these models are particularly attractive in the new system pharmacology paradigm, which focuses on gene-gene interaction networks rather than a single therapeutic target [9]. However, they still face major challenges, such as poor transferability, complex reward function optimization, and integration of complex omics data [43]. Recent examples where multiomics data were integrated and evaluated using an AI driven approach have been reported describing novel therapeutic targets [44–46].

Single cell and spatial transcriptomic analysis could be implemented in the future for target identification with the inclusion of AI techniques. For example, automated pattern recognition can be employed for image analysis in spatial transcriptomics to detect histological patterns and link them with gene expression data [47]. This combination of spatial data with histopathological images provides a more comprehensive view of tissue biology. CNNs are particularly useful in automating the detection of these spatial patterns [48]. Similarly, mutational patterns can be recognized by imaging techniques to distinguish distinct phenotypes in solid tumors [49, 50].

#### Machine learning for predicting druggability

Druggability refers to the likelihood that a biological target can be modulated by a small molecule or biological drug. AI models, particularly supervised ML algorithms, can be trained on datasets to allow for differentiation of known druggable and non-druggable targets​. These features may include structural properties of the protein, its function, interaction partners, and its role in disease pathways [30].

Structure-based drug design (SBDD) relies heavily on knowing the 3D structure of a protein to design molecules that can bind effectively to the target. Till very recently, druggability prediction was limited by the availability of experimentally determined protein structures, through X-ray crystallography or cryogenic electron microscopy (cryo-EM) [51]. Recent initiatives such as AlphaFold have revolutionized life sciences by predicting over 200 million protein structures available in the AlphaFold DataBase [52, 53]. This number far exceeds the number of experimentally resolved protein structures entries in the Protein Data Bank [54]. In addition, AlphaFold has demonstrated unprecedented accuracy, taking first place in the 14th edition of the biennial Critical Assessment of Structure Prediction (CASP) competition [55]. The revolution in structure prediction brought about by AI algorithms has greatly advanced SBDD strategies. These tools now leverage AlphaFold’s capabilities to uncover binding sites, key structural features, and protein interaction surfaces on therapeutic targets, enabling more efficient drug design campaigns [52]. In recent years, neural network models have been developed at a variety of scales —such as 3D structure, atomic interactions, or protein surfaces— to identify potential binding sites in therapeutical targets [56]. These advances allow the exploration of novel regions within complex proteins and open new opportunities for drug discovery.

### Drug discovery with artificial intelligence

Traditionally, computer-aided drug design (CADD) involves computational approaches to predict the interaction of drugs with biological targets [57]. The following section explores how AI can be utilized across different stages of drug discovery and highlights some of the most successful AI-driven innovations in the field.

#### Hit-to-lead approaches

***AI in virtual screening.*** Virtual screening is a crucial step in drug discovery where large chemical libraries are analyzed computationally to identify compounds that are most likely to interact with a specific biological target [58]. For structure-based virtual screening (SBVS), the 3D structure of the target is used to predict how different compounds will bind to the selected pocket. This method requires detailed knowledge of the target’s binding site [57]. Traditionally, docking simulations involved generating multiple poses of a molecule and calculating their binding energy scores. Yet, CADD is still far from being optimal, and pharma companies use conventional wet lab screening strategies to identify drugs for the targeting of a particular protein.

ML models have been used for decades in ligand-based virtual screening (LBVS) strategies, where properties (or descriptors) of known ligands for a given target are used to explore new candidates through predictive models known as Quantitative Structure-Activity Relationships (QSAR) [59]. However, AI revolution in drug discovery applied to QSAR is relatively recent, benefiting from new molecular representations and DL architectures. As a result, so-called deep QSAR allows for more efficient screening of ultra-large compound libraries, which can be combined with virtual screening techniques such as pharmacophore modeling or molecular docking [60]. The latter has been widely used in SBVS strategies, which rely on knowledge of the 3D structure of the target protein and compounds to identify potential inhibitor molecules. In this field, AI models have led to improvements in classification methods, binding pocket discovery, and scoring functions to evaluate ligand-protein binding affinity [61]. Much effort is being invested in the development of novel scoring functions as they can also contribute to other aspects of drug design such as lead optimization, prediction of absorption, distribution, metabolism, excretion and toxicity (ADMET) properties, and even QSAR models. In fact, ML models have demonstrated better performance compared to traditional scoring functions [62]. Emerging DL-based scoring functions, especially CNN models, are becoming established in virtual screening [63]. These models can process large amounts of data and recognize patterns in chemical structures that correlate with successful binding to biological targets. As more high-quality experimental data becomes publicly available, DL scoring functions are likely to gradually replace traditional ML approaches.

***De Novo drug design with AI.*** One of the most transformative applications of AI in drug discovery is de novo drug design, where AI models are used to generate entirely new molecular structures that have never been synthesized before. As mentioned, traditional methods of drug discovery depend on existing chemical libraries, but AI allows for the creation of novel compounds optimized for specific biological properties [64]. RL approach is used in de novo drug design, helping to iteratively improve the design of molecules by receiving feedback on each iteration’s success in meeting specific criteria like binding affinity, stability or pose [65, 66]. Generative models including GANs and variational autoencoders (VAEs) are used in creating new chemical structures by learning from existing data [67]. These models are particularly useful in expanding chemical libraries with the aim to create novel chemical space. However, the main limitations are that the newly created molecules could not be stable, synthesizable, or biologically active.

#### AI in lead optimization

Lead optimization is a critical phase of drug discovery where the structure of a lead compound is refined to improve its efficacy, reduce toxicity, and enhance its pharmacokinetic properties. AI can help in lead optimization by predicting how modifications to a molecular structure will affect its overall drug-like properties [68].

***Molecular dynamics (MD) simulations with AI.*** AI enhances MD simulations by providing more accurate predictions of how molecules will behave in different physiological environments. MD simulations are used to predict the stability of a drug when bound to its target, as well as how the drug will interact with biological membranes, enzymes, or transporters [69]​. AI-based models accelerate these simulations, making it possible to simulate complex biological interactions in a fraction of the time it would take using traditional methods [70, 71]​.

***Predicting drug-drug interactions and toxicity.*** In traditional drug discovery, predicting the ADMET properties of a compound is time-consuming and costly [72]. AI models can predict the potential for drug-drug interactions and adverse reactions by analyzing molecular structures and biological pathways. By integrating data from chemical structures, biological activity, and known drug interactions, AI models can identify compounds that are more likely to cause toxicity or other adverse effects in patients [73–75]​. Additionally, it can allow earlier discontinuation of development of candidates with unfavorable profiles​ therefore reducing the probability of failure due to toxicity in early clinical trials [76].

***Use of multimodal models and QSAR in drug discovery***: Multimodal models integrate multiple types of data (e.g., chemical information, biological data, and several other parameters) into a single framework for more comprehensive understanding [77]. Multimodal models can also be integrated into QSAR models. These models are used in drug discovery to predict the biological activity of chemical compounds based on their chemical structure [78]. QSAR modeling involves correlating structural features of molecules including functional groups, molecular weight, or polarity, with their observed biological activity [79]. AI aims to significantly enhance QSAR methods, allowing the development of more accurate and predictive models.

AI-driven QSAR models use ML algorithms such as random forests, support vector machines (SVMs), and neural networks to learn from large databases of molecular structures and their corresponding activities, generating highly accurate predictions for new compounds [80]. For example, random forests are particularly useful for handling datasets with irrelevant features, while neural networks can model complex, non-linear relationships between descriptors and biological activity [81]​. Deep neural networks (DNNs) are capable of learning hierarchical features from raw molecular data, allowing for the automatic discovery of relevant molecular descriptors without the need for manual feature engineering [82]​. Additionally, CNNs have been applied to molecular graphs, where molecules are represented as nodes (atoms) and edges (bonds). CNNs can learn from these graph representations to predict properties such as binding affinity and toxicity​. This approach, often referred to as graph-based QSAR, leverages the structural information embedded in chemical graphs to enhance the predictive power of QSAR models [77]​.

An example of the integration of biological data for drug identification is PaccMann, an AI-driven framework designed to predict cancer cell sensitivity to compounds by integrating molecular structures, gene expression profiles, and protein interaction data. It utilizes attention-based neural networks to forecast drug efficacy and highlight key biological factors influencing predictions [83]. An extension, PaccMann^RL, employs RL to generate novel anticancer compounds tailored to specific cancer transcriptomic profiles. By conditioning drug design on gene expression data, PaccMann^RL enables personalized therapy development [84]. These AI-based approaches enhance anticancer drug discovery by improving precision, interpretability, and treatment optimization.

### AI in precision medicine and early clinical development

Biomarkers are biological indicators that can predict outcome (prognostic biomarkers) and/or help identify which patients are most likely to benefit from a specific drug (predictive biomarkers) [85, 86]. AI can analyze genomic, proteomic, and clinical data to identify novel biomarkers associated with drug response or resistance​. By predicting which patients are likely to respond positively to a drug based on their genetic profile, AI can facilitate the development of personalized treatment strategies [87].

AI is used increasingly to develop personalized medicine strategies. ML algorithms are employed to analyze genetic data from tumor biopsies, enabling the identification of targeted therapies that are most likely to be effective for individual patients [36]. Similarly, AI technologies applied to histology could be used to evaluate the origin of cancers from unknown primary sites [88]. However, just like with traditional biomarker development and validation, AI tools need data from comparative studies to be able to differentiate between prognostic and predictive biomarkers. Similarly, the ability of AI to link different data into networks can enhance the capability to simulate comparative studies thereby aiding in this process.

AI can be used in different areas from early drug development including: (1) Patient recruitment and site selection. AI algorithms can assess patient eligibility more quickly by evaluating electronic health records (EHRs), ensuring suitable candidates are screened for trials. (2) Predictive modeling for trial outcomes including dose escalation in early clinical studies or prediction of toxicity. AI can simulate different trial designs by predicting potential outcomes based on historical data, trial parameters, and patient characteristics [89]. AI-based predictive models can help reduce trial failures by focusing on trial designs with the highest likelihood of success [37]. (3) Protocol optimization. AI tools can allow for optimization of trial protocols by simulating different scenarios and adjusting for variables like dosage, treatment duration, and patient characteristics [37]. (4) Adaptive Trial Designs: AI can enable adaptive trial designs where data from ongoing trials are continuously analyzed. This allows modifications to be made in real time, such as adjusting dosages or patient cohorts based on interim results, ultimately increasing the efficiency and success rate of studies [90]. This also includes decentralized clinical trials (DCTs) where AI enables decentralized trials by supporting remote monitoring, virtual visits, and digital data collection [91]. (5) Synthetic control arms (SCAs) are a groundbreaking innovation in clinical trial design, enabled largely by AI and advanced data analytics. In SCAs instead of recruiting additional participants for a placebo or control group, real-world data (RWD) and historical trial data is used to simulate the outcomes of a control arm. This approach addresses ethical, logistical, and cost-related challenges in traditional trials [91, 92]. (6) Digital Twins. The creation of digital replicas of patients allows for the testing of treatments in a virtual environment before actual application, reducing risks and optimizing therapeutic strategies [93, 94].

Table 1 provides a list of areas with their applications and limitations.

**Table 1:** AI in clinical development and trial design, description of application and potential limitations

Area	Applications	Limitations
**1. Patient Recruitment and Site Selection**	AI algorithms evaluate EHRs to quickly assess patient eligibility, ensuring suitable candidates are screened for trials.	Relies on access to high-quality, standardized EHRs. Privacy concerns and potential biases in the data can affect recruitment accuracy.
**2. Predictive Modeling for Trial Outcomes**	AI simulates different trial designs, including dose escalation in early clinical studies and prediction of toxicity, using historical data, trial parameters, and patient characteristics. Predictive models focus on trial designs with the highest likelihood of success.	Predictions may not fully capture real-world complexities. Dependence on historical data may limit generalizability for novel treatments.
**3. Protocol Optimization**	AI tools simulate different trial scenarios, optimizing protocols by adjusting variables like dosage, treatment duration, and patient characteristics.	Optimization outcomes are only as good as the input data and models. Over-reliance on AI could overlook nuanced clinical or ethical considerations.
**4. Adaptive Trial Designs**	Enables ongoing data analysis to make real-time modifications to trials, such as adjusting dosages or patient cohorts based on interim results. Supports DCTs by facilitating remote monitoring, virtual visits, and digital data collection.	Real-time adjustments require robust infrastructure and regulatory approval processes. Decentralized trials depend on patient access to reliable digital technology.
**5. Synthetic Control Arms (SCAs)**	Uses RWD and historical trial data to simulate control arms, reducing the need for placebo groups. Addresses ethical, logistical, and cost challenges in traditional trials.	SCAs depend on high-quality, well-annotated datasets. Historical data may not fully represent the current trial population. Regulatory acceptance is still evolving.
**6. Digital Twins**	Creates digital replicas of patients, allowing virtual testing of treatments before application. Reduces risks and optimizes therapeutic strategies.	Developing accurate digital twins is complex and computationally intensive. Requires detailed patient data, raising privacy and data-sharing concerns.

For early clinical trial development researchers have developed explainable AI methods using natural language processing to enhance patient matching for Phase I oncology clinical trials, addressing significant challenges in patient recruitment and improving efficiency in drug development. By leveraging AI-driven tools, these methods analyze complex clinical trial eligibility criteria and patient records, ensuring that suitable candidates are identified more accurately and efficiently [95, 96]. Additionally, AI is increasingly being used to manage clinical trials, streamlining key tasks such as writing protocols, recruiting patients, and analyzing trial data [97].

In addition to the previously described, the future goal is that early trial designs will not only be restricted to a classical dose escalation design in which safety and biological signs of target engagement are evaluated, but also to define better clinical development strategies. This approach can also align with current regulatory strategies like the US Food and Drug Administration’s (FDA) Project Optimus which seeks to reform the dose optimization and dose selection paradigm in oncology drug development. This change is aimed at minimizing unnecessary toxicity while maximizing therapeutic benefits. Indeed, this initiative promotes early dose-finding studies and requires drug developers to characterize the dose-exposure, pharmacodynamic, and toxicity relationships more thoroughly across different doses before progressing to registration trials [98]. In summary, AI-driven approaches can be integrated across various stages of early drug development, accelerating timelines and enhancing accuracy in patient selection and clinical advancement.

### Limitations of AI applied to early drug discovery and development

There are several limitations for the application of AI to early drug discovery and development. Some of them can be grouped as computational, ethical/regulatory or those related to data quality. From a computational and data quality point of view, AI models can exhibit bias if the training data is not representative of the population or if data are limited (such as absence of comparative analyses). For example, if the data used to train models is disproportionately from certain demographics, the resulting predictions may not be accurate for underrepresented groups, which can lead to suboptimal drug development outcomes. Indeed, bias in training datasets poses a significant challenge in the clinical development of new cancer drugs, affecting predictive accuracy, patient selection, and treatment efficacy. Many oncology clinical trials predominantly enroll patients of European ancestry, despite the diverse genetic backgrounds of cancer patients worldwide [99]. AI models trained on biased datasets can perform worse when applied to previously unseen populations [100]. To mitigate these challenges, synthetic data (SD) generation is increasingly being explored as a solution to balance underrepresented patient groups in training datasets [100, 101]. In line with this, but in the drug discovery space, many AI models rely on historical datasets, which may be incomplete, inconsistent, or not representative of new drug targets or chemical entities.

The use of SD can help to increase the available information. SD includes artificially generated information that mimics RWD while not directly originating from actual observations or measurements [102, 103]. SD generation leverages AI methods such as GANs, VAEs, transformers, and diffusion models to create realistic datasets (Table 2). Synthetic biological data, such as omics-based datasets, allow researchers to model disease progression and drug responses virtually. For instance, the conditional single-cell Generative Adversarial Network (cscGAN) generates realistic single-cell RNA sequencing (scRNA-seq) data, enabling researchers to study gene expression patterns associated with diseases like cancer and neurodegenerative disorders [104]. Additionally, DeepNovo, a DL-based tool, synthetically generates peptide spectra, improving mass spectrometry-based protein sequencing for proteomics applications [105, 106]. Similarly, DeepDock (version Dock2D-IP and Dock2D-IF), an AI-driven molecular docking approach, utilizes synthetic protein-ligand interaction data to predict binding affinities with high accuracy, facilitating virtual screening and lead optimization [107].

**Table 2:** AI methodologies and their application in drug design including limitations in drug discovery

Topic	Definition	Challenges in Drug Discovery
**Generative Adversarial Networks (GANs)**	GANs consist of two neural networks: a generator, which creates new molecular structures, and a discriminator, which evaluates these structures for drug-like properties.	- Ensuring generated molecules are chemically valid and synthesizable. - Training requires large datasets of molecular interactions to improve accuracy.
**Variational Autoencoders (VAEs)**	VAEs are generative models that encode molecular data into a latent space and decode new molecules from this space, exploring chemical variations and optimizing lead compounds.	- The quality of the latent space heavily influences the diversity of generated molecules. - Difficulty in generating highly diverse chemical libraries.
**Reinforcement Learning (RL)**	RL involves an agent that explores the chemical space, receiving rewards for actions that improve drug-like properties such as binding affinity, solubility, and toxicity.	- Defining effective reward functions to balance multiple objectives. - High computational resources are required for effective training and exploration.
**Convolutional Neural Networks (CNNs)**	CNNs are deep learning models that use convolutional layers to extract spatial features from data, often used to predict binding affinities and interactions between ligands and proteins.	- CNNs require large, high-quality 3D molecular datasets to train effectively. - Balancing speed and accuracy in large-scale virtual screening.
**Combination of GANs**,** RL**,** and CNNs**	Combining GANs to generate novel molecules, CNNs to assess protein-ligand interactions, and RL to refine drug candidates optimizes multiple properties in de novo drug design.	- Integrating these models requires large datasets and computational power. - Balancing multiple objectives, such as efficacy, safety, and synthesizability.

SD also has overfitting risks as models trained extensively on SD may not generalize well if it is too idealized or lacks real-world variability [103]. As is the case with more basic computational models, the greater the sensitivity and specificity of input data, the more robust the output of AI models can be. Regarding regulatory concerns, AI-driven drug discovery often requires access to patient data, raising privacy issues ensuring compliance with data protection regulations [37]. In addition, regulatory bodies like the FDA require substantial evidence for safety and efficacy, which may not align with AI’s rapid, iterative predictions. Table 3 provides a comprehensive summary of the challenges and limitations described before. In summary, although challenges remain in applying AI to early drug discovery and development, its continuous integration across various processes is expected to gradually mitigate these limitations.

**Table 3:** Comprehensive view of the computational, ethical/regulatory, and data quality challenges facing AI in early drug discovery and development

Area		Challenges	Examples
**Computational**	**Bias in AI Models**	AI models may exhibit bias if the training data is not representative of the entire population, leading to inaccurate predictions for underrepresented groups.	For example, a dataset heavily weighted toward certain demographics could result in models that fail to predict drug efficacy or safety for other populations. This bias can lead to suboptimal drug development and approval challenges.
	**Reliance on Historical Data**	Many AI models rely on historical datasets, which may be incomplete, inconsistent, or not representative of novel drug targets or chemical entities.	Historical datasets may lack modern advances in understanding disease biology, leading to inaccuracies. This can result in inefficiencies or missed opportunities in identifying new therapeutic targets.
	**Use of Synthetic Data**	SD artificially mimics real-world data and can expand training datasets, especially when RMW is limited.	While SD can increase data availability, overfitting risks arise when models trained extensively on synthetic data fail to generalize to real-world scenarios. SD may also lack real-world variability, leading to overly idealized outcomes.
	**Dependence on Input Data Sensitivity and Specificity**	The quality and robustness of AI predictions are directly tied to the sensitivity and specificity of the input data used for model training.	Low-quality input data leads to unreliable model outputs, undermining the effectiveness of AI applications. Accurate predictions depend on high-quality, comprehensive, and representative datasets.
**Ethical/** **Regulatory**	**Data Privacy and Security Concerns**	AI-driven drug discovery requires access to large volumes of patient data, raising concerns about data privacy and compliance with data protection regulations.	Ensuring adherence to laws and regulations is critical. Mismanagement of sensitive patient data can result in legal consequences.
	**Regulatory Evidence Requirements**	Regulatory bodies, such as the FDA, demand substantial evidence for drug safety and efficacy, which may not align with AI’s iterative, predictive nature.	AI models often produce probabilistic predictions that may lack the traditional evidence base required by regulatory authorities. This mismatch can slow drug approval processes and limit the adoption of AI-driven insights in regulatory submissions.
**Data Quality**	**Incomplete and Inconsistent Datasets**	Historical datasets may lack critical variables, include outdated information, or be inconsistent across sources.	These issues reduce the reliability of AI models in identifying effective drug candidates or predicting outcomes, especially when training data is missing key aspects of newer drug targets or modalities.
	**Limited Representativeness**	Datasets may not adequately represent diverse patient populations, leading to suboptimal outcomes in drug discovery and development.	For instance, AI models trained on data predominantly from one geographic region may fail to generalize findings to other regions or ethnic groups.
	**Overfitting Risks in Synthetic Data**	Models trained excessively on synthetic data may struggle to generalize to real-world scenarios if the SD lacks variability or is overly idealized.	For example, a model trained only on synthetic datasets with perfectly curated characteristics may fail to handle the messy, variable nature of real-world patient data.
	**Integration of Multimodal** **Data Sources**	Combining structured (e.g., lab results) and unstructured data (e.g., clinical notes) remains challenging, especially in ensuring compatibility and consistency across formats.	Without effective integration, AI models risk missing critical insights or generating incomplete analyses, reducing their utility in drug development pipelines.

## Conclusions

AI offers significant advantages in addressing the challenges of classical drug discovery and development. AI can analyze large datasets for target identification, optimize chemical leads, and improve efficiency in virtual screening. It also aids in early clinical trials by enhancing patient recruitment and predicting outcomes to reduce trial failures. In personalized medicine, AI can help discover the difference between simple prognostic biomarkers and those that predict patient responses to treatments, streamlining cancer therapy development and improving success rates. However, there are still limitations that cannot be improved with the use of AI. For instance, AI cannot help to predict the use of inadequate preclinical models used in preclinical research. Many preclinical assays do not accurately represent the complexities of human tumors. As a result, some drugs that perform well in simplified models fail when tested in more complex human systems [108]. This has been clearly observed with immune oncology agents.

In summary AI can be a highly valuable tool if correctly applied to several of the drug discovery and development processes. However, integration with current models will be challenging and time consuming; with the incorporation of multitasker teams, AI tools could boost the drug development process.

## Data Availability

No datasets were generated or analysed during the current study.

## Abbreviations

AI: Artificial Intelligence
HTS: High-Throughput Screening
SAR: Structure-Activity Relationship
MTD: Maximum Tolerated Dose
DLT: Dose-Limiting Toxicity
DL: Deep Learning
ML: Machine Learning
CNN: Convolutional Neural Network
RNN: Recurrent Neural Network
GAN: Generative Adversarial Network
RL: Reinforcement Learning
SBDD: Structure-based Drug Design
cryo-EM: Cryogenic Electron Microscopy
CASP: Critical Assessment of Structure Prediction
CADD: Computer-Aided Drug Design
SBVS: Structure-based Virtual Screening
LBVS: Ligand-based Virtual Screening
QSAR: Quantitative Structure-Activity Relationship
ADMET: Absorption, Distribution, Metabolism, Excretion, and Toxicity
VAE: Variational Autoencoders
MD: Molecular Dynamics
SVM: Support Vector Machines
DNN: Deep Neural Network
EHR: Electronic Health Record
DCT: Decentralized Clinical Trial
SCA: Synthetic Control Arm
RWD: Real-World Data
FDA: Food and Drug Administration
SD: Synthetic Data
cscGAN: conditional single-cell Generative Adversarial Network
scRNA-seq: Single-cell RNA sequencing

## References

[1] Accelerating biopharmaceutical development while reducing costs| McKinsey. https://www.mckinsey.com/industries/life-sciences/our-insights/the-pursuit-of-excellence-in-new-drug-development

[2] Wouters OJ, McKee M, Luyten J. Estimated research and development investment needed to bring a new medicine to market, 2009–2018. JAMA. 2020;323:844–53. 32125404 10.1001/jama.2020.1166 PMC7054832

[3] Paul SM, et al. How to improve R&D productivity: the pharmaceutical industry’s grand challenge. Nat Rev Drug Discov. 2010;9:203–14. 20168317 10.1038/nrd3078

[4] Murmu A, Győrffy B. Artificial intelligence methods available for cancer research. Front Med. 2024;18:778–97. 39115792 10.1007/s11684-024-1085-3

[5] Ocana A, Pandiella A, Siu LL, Tannock IF. Preclinical development of molecular-targeted agents for cancer. Nat Rev Clin Oncol. 2011;8:200–9. 10.1038/nrclinonc.2010.194 21135887

[6] Corrales Sánchez V, et al. Screening and preliminary biochemical and biological studies of [RuCl(p-cymene)(N,N-bis(diphenylphosphino)-isopropylamine)][BF4] in breast Cancer models. ACS Omega. 2019;4:13005–14. 31460427 10.1021/acsomega.9b00296 PMC6704442

[7] Aldea M et al. Precision medicine in the era of multi-omics: can the data tsunami guide rational treatment decision? ESMO Open 8, (2023). 10.1016/j.esmoop.2023.101642 PMC10539962 37769400

[8] Amir E, et al. Oncogenic targets, magnitude of benefit, and market pricing of antineoplastic drugs. JCO. 2011;29:2543–9. 10.1200/JCO.2011.35.2393 21606435

[9] Ocaña A, Pandiella A. Personalized therapies in the cancer ‘omics’ era. Mol Cancer. 2010;9:202. 20670437 10.1186/1476-4598-9-202 PMC2920264

[10] Chang L, et al. Systematic profiling of conditional pathway activation identifies context-dependent synthetic lethalities. Nat Genet. 2023;55:1709–20. 37749246 10.1038/s41588-023-01515-7

[11] Shalem O, Sanjana NE, Zhang F. High-throughput functional genomics using CRISPR–Cas9. Nat Rev Genet. 2015;16:299–311. 25854182 10.1038/nrg3899 PMC4503232

[12] Vazquez F, Sellers WR. Are CRISPR screens providing the next generation of therapeutic targets?? Cancer Res. 2021;81:5806–9. 34853037 10.1158/0008-5472.CAN-21-1784 PMC10078623

[13] Wang T, et al. Identification and characterization of essential genes in the human genome. Science. 2015;350:1096–101. 26472758 10.1126/science.aac7041 PMC4662922

[14] Tiedt R et al. Integrated CRISPR screening and drug profiling identifies combination opportunities for EGFR, ALK, and BRAF/MEK inhibitors. Cell Rep 42, (2023). 10.1016/j.celrep.2023.112297 36961816

[15] Li R, et al. Comparative optimization of combinatorial CRISPR screens. Nat Commun. 2022;13:2469. 35513429 10.1038/s41467-022-30196-9 PMC9072436

[16] Pech MF, et al. Systematic identification of cancer cell vulnerabilities to natural killer cell-mediated immune surveillance. eLife. 2019;8:e47362. 31452512 10.7554/eLife.47362 PMC6713475

[17] Sulahian R, et al. Synthetic lethal interaction of SHOC2 depletion with MEK Inhibition in RAS-Driven cancers. Cell Rep. 2019;29:118–e1348. 31577942 10.1016/j.celrep.2019.08.090 PMC6918830

[18] Kryukov GV, et al. MTAP deletion confers enhanced dependency on the PRMT5 arginine methyltransferase in cancer cells. Science. 2016;351:1214–8. 26912360 10.1126/science.aad5214 PMC4997612

[19] MTAP Deletion Promotes. Cancer-Cell dependence on PRMT5. Cancer Discov. 2016;6:OF11.

[20] Rask-Andersen M, Almén MS, Schiöth HB. Trends in the exploitation of novel drug targets. Nat Rev Drug Discov. 2011;10:579–90. 21804595 10.1038/nrd3478

[21] Imming P, Sinning C, Meyer A. Drugs, their targets and the nature and number of drug targets. Nat Rev Drug Discov. 2006;5:821–34. 17016423 10.1038/nrd2132

[22] Hopkins AL, Groom CR. The druggable genome. Nat Rev Drug Discov. 2002;1:727–30. 12209152 10.1038/nrd892

[23] Arkin MR, Wells JA. Small-molecule inhibitors of protein–protein interactions: progressing towards the dream. Nat Rev Drug Discov. 2004;3:301–17. 15060526 10.1038/nrd1343

[24] Nevola L, Giralt E. Modulating protein–protein interactions: the potential of peptides. Chem Commun. 2015;51:3302–15. 10.1039/c4cc08565e 25578807

[25] Macarron R, et al. Impact of high-throughput screening in biomedical research. Nat Rev Drug Discov. 2011;10:188–95. 21358738 10.1038/nrd3368

[26] Liew SK, Malagobadan S, Arshad NM, Nagoor NH. A review of the Structure–Activity relationship of natural and synthetic antimetastatic compounds. Biomolecules. 2020;10:138. 31947704 10.3390/biom10010138 PMC7022821

[27] Benitez JC, et al. Late phase 1 studies: concepts and outcomes. Lancet Oncol. 2021;22:e446–55. 34592194 10.1016/S1470-2045(21)00467-8

[28] Eisenhauer EA, O’Dwyer PJ, Christian M, Humphrey JS. Phase I clinical trial design in Cancer drug development. JCO. 2000;18:684–684. 10.1200/JCO.2000.18.3.684 10653884

[29] Manji A, et al. Evolution of clinical trial design in early drug development: systematic review of expansion cohort use in Single-Agent phase I Cancer trials. JCO. 2013;31:4260–7. 10.1200/JCO.2012.47.4957 24127441

[30] Chan HCS, Shan H, Dahoun T, Vogel H, Yuan S. Advancing drug discovery via artificial intelligence. Trends Pharmacol Sci. 2019;40:592–604. 31320117 10.1016/j.tips.2019.06.004

[31] Cerchia C, Lavecchia A. New avenues in artificial-intelligence-assisted drug discovery. Drug Discovery Today. 2023;28:103516. 36736583 10.1016/j.drudis.2023.103516

[32] Chang L, Ruiz P, Ito T, Sellers WR. Targeting pan-essential genes in cancer: challenges and opportunities. Cancer Cell. 2021;39:466–79. 33450197 10.1016/j.ccell.2020.12.008 PMC8157671

[33] Alcaraz-Sanabria A, et al. Synthetic lethality interaction between Aurora kinases and CHEK1 inhibitors in ovarian Cancer. Mol Cancer Ther. 2017;16:2552–62. 28847989 10.1158/1535-7163.MCT-17-0223

[34] Rudolph J, Settleman J, Malek S. Emerging trends in Cancer drug Discovery—From drugging the undruggable to overcoming resistance. Cancer Discov. 2021;11:815–21. 33811118 10.1158/2159-8290.CD-21-0260

[35] Menyhárt O, Győrffy B. Multi-omics approaches in cancer research with applications in tumor subtyping, prognosis, and diagnosis. Comput Struct Biotechnol J. 2021;19:949–60. 33613862 10.1016/j.csbj.2021.01.009 PMC7868685

[36] Esteva A, et al. A guide to deep learning in healthcare. Nat Med. 2019;25:24–9. 30617335 10.1038/s41591-018-0316-z

[37] Topol EJ. High-performance medicine: the convergence of human and artificial intelligence. Nat Med. 2019;25:44–56. 30617339 10.1038/s41591-018-0300-7

[38] You Y, et al. Artificial intelligence in cancer target identification and drug discovery. Sig Transduct Target Ther. 2022;7:1–24. 10.1038/s41392-022-00994-0 PMC9090746 35538061

[39] Camacho DM, Collins KM, Powers RK, Costello JC, Collins JJ. Next-Generation machine learning for biological networks. Cell. 2018;173:1581–92. 29887378 10.1016/j.cell.2018.05.015

[40] do Valle ÍF, et al. Network integration of multi-tumour omics data suggests novel targeting strategies. Nat Commun. 2018;9:4514. 30375513 10.1038/s41467-018-06992-7 PMC6207774

[41] Muzio G, O’Bray L, Borgwardt K. Biological network analysis with deep learning. Brief Bioinform. 2021;22:1515–30. 33169146 10.1093/bib/bbaa257 PMC7986589

[42] Zhao L, Wang J, Pang L, Liu Y, Zhang J. GANsDTA: predicting Drug-Target binding affinity using GANs. Front Genet 10, (2020). 10.3389/fgene.2019.01243 PMC6962343 31993067

[43] Tan RK, Liu Y, Xie L. Reinforcement learning for systems pharmacology-oriented and personalized drug design. Expert Opin Drug Discov. 2022;17:849–63. 35510835 10.1080/17460441.2022.2072288 PMC9824901

[44] Kamya P, et al. PandaOmics: an AI-Driven platform for therapeutic target and biomarker discovery. J Chem Inf Model. 2024;64:3961–9. 38404138 10.1021/acs.jcim.3c01619 PMC11134400

[45] Ren F, et al. A small-molecule TNIK inhibitor targets fibrosis in preclinical and clinical models. Nat Biotechnol. 2025;43:63–75. 38459338 10.1038/s41587-024-02143-0 PMC11738990

[46] Chen RJ, et al. Pathomic fusion: an integrated framework for fusing histopathology and genomic features for Cancer diagnosis and prognosis. IEEE Trans Med Imaging. 2022;41:757–70. 32881682 10.1109/TMI.2020.3021387 PMC10339462

[47] De Zuani M, et al. Single-cell and Spatial transcriptomics analysis of non-small cell lung cancer. Nat Commun. 2024;15:4388. 38782901 10.1038/s41467-024-48700-8 PMC11116453

[48] Asp M, Bergenstråhle J, Lundeberg J. Spatially resolved Transcriptomes—Next generation tools for tissue exploration. BioEssays. 2020;42:1900221. 10.1002/bies.201900221 32363691

[49] Fu Y, et al. Pan-cancer computational histopathology reveals mutations, tumor composition and prognosis. Nat Cancer. 2020;1:800–10. 35122049 10.1038/s43018-020-0085-8

[50] Rigamonti A, et al. Integrating AI-Powered digital pathology and imaging mass cytometry identifies key classifiers of tumor cells, stroma, and immune cells in Non–Small cell lung Cancer. Cancer Res. 2024;84:1165–77. 38315789 10.1158/0008-5472.CAN-23-1698 PMC10982643

[51] Cheng Y. Single-Particle Cryo-EM at crystallographic resolution. Cell. 2015;161:450–7. 25910205 10.1016/j.cell.2015.03.049 PMC4409662

[52] Jumper J, et al. Highly accurate protein structure prediction with alphafold. Nature. 2021;596:583–9. 34265844 10.1038/s41586-021-03819-2 PMC8371605

[53] Varadi M, et al. AlphaFold protein structure database in 2024: providing structure coverage for over 214 million protein sequences. Nucleic Acids Res. 2024;52:D368–75. 37933859 10.1093/nar/gkad1011 PMC10767828

[54] Berman HM, et al. The protein data bank. Nucleic Acids Res. 2000;28:235–42. 10592235 10.1093/nar/28.1.235 PMC102472

[55] AlphaFold. a solution to a 50-year-old grand challenge in biology. *Google DeepMind* https://deepmind.google/discover/blog/alphafold-a-solution-to-a–50-year-old-grand-challenge-in-biology/ (2025).

[56] Geraseva EP. Deep learning methods for binding site prediction in protein structures. Biochem Mosc Suppl Ser B. 2024;18:103–17.

[57] Lionta E, Spyrou G, Vassilatis DK, Cournia Z. Structure-Based virtual screening for drug discovery: principles, applications and recent advances. Curr Top Med Chem 14, 1923–38. 10.2174/1568026614666140929124445 PMC4443793 25262799

[58] Jorgensen WL. The many roles of computation in drug discovery. Science. 2004;303:1813–8. 15031495 10.1126/science.1096361

[59] Meli R, Morris GM, Biggin PC. Scoring functions for Protein-Ligand binding affinity prediction using Structure-based deep learning: A review. Front Bioinform 2, (2022). 10.3389/fbinf.2022.885983 PMC7613667 36187180

[60] Tropsha A, Isayev O, Varnek A, Schneider G, Cherkasov A. Integrating QSAR modelling and deep learning in drug discovery: the emergence of deep QSAR. Nat Rev Drug Discov. 2024;23:141–55. 38066301 10.1038/s41573-023-00832-0

[61] Crampon K, Giorkallos A, Deldossi M, Baud S, Steffenel LA. Machine-learning methods for ligand–protein molecular Docking. Drug Discovery Today. 2022;27:151–64. 34560276 10.1016/j.drudis.2021.09.007

[62] Shen C, et al. From machine learning to deep learning: advances in scoring functions for protein–ligand Docking. WIREs Comput Mol Sci. 2020;10:e1429.

[63] McNutt AT, et al. GNINA 1.0: molecular Docking with deep learning. J Cheminform. 2021;13:43. 34108002 10.1186/s13321-021-00522-2 PMC8191141

[64] Schneider G. Automating drug discovery. Nat Rev Drug Discov. 2018;17:97–113. 29242609 10.1038/nrd.2017.232

[65] Popova M, Isayev O, Tropsha A. Deep reinforcement learning for de Novo drug design. Sci Adv. 2018;4:eaap7885. 30050984 10.1126/sciadv.aap7885 PMC6059760

[66] Fang Y, Pan X, Shen H-B. De Novo drug design by iterative multiobjective deep reinforcement learning with graph-based molecular quality assessment. Bioinformatics. 2023;39:btad157. 36961341 10.1093/bioinformatics/btad157 PMC10085518

[67] Sanchez-Lengeling B, Aspuru-Guzik A. Inverse molecular design using machine learning: generative models for matter engineering. Science. 2018;361:360–5. 30049875 10.1126/science.aat2663

[68] de Souza Neto LR et al. In Silico strategies to support Fragment-to-Lead optimization in drug discovery. Front Chem 8, (2020). 10.3389/fchem.2020.00093 PMC7040036 32133344

[69] Noé F, Tkatchenko A, Müller K-R, Clementi C. Machine learning for molecular simulation. Annu Rev Phys Chem. 2020;71:361–90. 32092281 10.1146/annurev-physchem-042018-052331

[70] Chmiela S, et al. Machine learning of accurate energy-conserving molecular force fields. Sci Adv. 2017;3:e1603015. 28508076 10.1126/sciadv.1603015 PMC5419702

[71] Zhang L, Han J, Wang H, Car R, E W. Deep potential molecular dynamics: A scalable model with the accuracy of quantum mechanics. Phys Rev Lett. 2018;120:143001. 29694129 10.1103/PhysRevLett.120.143001

[72] Di L, Kerns EH, Carter GT. Drug-Like property concepts in pharmaceutical design. Curr Pharm Design. 2009;15:2184–94. 10.2174/138161209788682479 19601822

[73] Bhhatarai B, Walters WP, Hop CECA, Lanza G, Ekins S. Opportunities and challenges using artificial intelligence in ADME/Tox. Nat Mater. 2019;18:418–22. 31000801 10.1038/s41563-019-0332-5 PMC6594826

[74] Ferreira LLG, Andricopulo A. D. ADMET modeling approaches in drug discovery. Drug Discovery Today. 2019;24:1157–65. 30890362 10.1016/j.drudis.2019.03.015

[75] Lin Z, Chou W-C. Machine learning and artificial intelligence in toxicological sciences. Toxicol Sci. 2022;189:7–19. 35861448 10.1093/toxsci/kfac075 PMC9609874

[76] Harrison RK. Phase II and phase III failures: 2013–2015. Nat Rev Drug Discovery. 2016;15:817–8. 27811931 10.1038/nrd.2016.184

[77] Kearnes S, McCloskey K, Berndl M, Pande V, Riley P. Molecular graph convolutions: moving beyond fingerprints. J Comput Aided Mol Des. 2016;30:595–608. 27558503 10.1007/s10822-016-9938-8 PMC5028207

[78] Cherkasov A, et al. QSAR modeling: where have you been?? where are you going to?? J Med Chem. 2014;57:4977–5010. 24351051 10.1021/jm4004285 PMC4074254

[79] Tropsha A. Best practices for QSAR model development, validation, and exploitation. Mol Inf. 2010;29:476–88. 10.1002/minf.201000061 27463326

[80] Chen H, Engkvist O, Wang Y, Olivecrona M, Blaschke T. The rise of deep learning in drug discovery. Drug Discovery Today. 2018;23:1241–50. 29366762 10.1016/j.drudis.2018.01.039

[81] Svetnik V, et al. Random forest: A classification and regression tool for compound classification and QSAR modeling. J Chem Inf Comput Sci. 2003;43:1947–58. 14632445 10.1021/ci034160g

[82] Mayr A, Klambauer G, Unterthiner T, Hochreiter S. DeepTox: toxicity prediction using deep learning. Front Environ Sci 3, (2016).

[83] Cadow J, Born J, Manica M, Oskooei A. Rodríguez Martínez, M. PaccMann: a web service for interpretable anticancer compound sensitivity prediction. Nucleic Acids Res. 2020;48:W502–8. 32402082 10.1093/nar/gkaa327 PMC7319576

[84] Born J, Manica M, Oskooei A, Cadow J, Rodríguez Martínez M. PaccMannRL: designing anticancer drugs from transcriptomic data via reinforcement learning In: Schwartz R, editor. Research in computational molecular biology. Cham: Springer International Publishing; 2020. pp. 231–3. 10.1007/978-3-030-45257-5-18. .

[85] Sawyers CL. The cancer biomarker problem. Nature. 2008;452:548–52. 18385728 10.1038/nature06913

[86] Henry NL, Hayes DF. Cancer biomarkers. Mol Oncol. 2012;6:140–6. 22356776 10.1016/j.molonc.2012.01.010 PMC5528374

[87] Libbrecht MW, Noble WS. Machine learning applications in genetics and genomics. Nat Rev Genet. 2015;16:321–32. 25948244 10.1038/nrg3920 PMC5204302

[88] Lu MY, et al. AI-based pathology predicts origins for cancers of unknown primary. Nature. 2021;594:106–10. 33953404 10.1038/s41586-021-03512-4

[89] Wason JMS, Jaki T. Optimal design of multi-arm multi-stage trials. Stat Med. 2012;31:4269–79. 22826199 10.1002/sim.5513

[90] Kairalla JA, Coffey CS, Thomann MA, Muller K. E. Adaptive trial designs: a review of barriers and opportunities. *Trials* 13, 145 (2012). 10.1186/1745-6215-13-145 PMC3519822 22917111

[91] Moingeon P, Chenel M, Rousseau C, Voisin E, Guedj M. Virtual patients, digital twins and causal disease models: paving the ground for *in Silico* clinical trials. Drug Discovery Today. 2023;28:103605. 37146963 10.1016/j.drudis.2023.103605

[92] Corrigan-Curay J, Sacks L, Woodcock J. Real-World evidence and Real-World data for evaluating drug safety and effectiveness. JAMA. 2018;320:867–8. 30105359 10.1001/jama.2018.10136

[93] Thorlund K, Dron L, Park JJ, Mills EJ. Synthetic and external controls in clinical trials: A primer for researchers. CLEP. 2020;12:457–67. 10.2147/CLEP.S242097 PMC7218288 32440224

[94] Bordukova M, Makarov N, Rodriguez-Esteban R, Schmich F, Menden MP. Generative artificial intelligence empowers digital twins in drug discovery and clinical trials. Expert Opin Drug Discov. 2024;19:33–42. 37887266 10.1080/17460441.2023.2273839

[95] Ghosh S, et al. Harnessing explainable artificial intelligence for patient-to-clinical-trial matching: A proof-of-concept pilot study using phase I oncology trials. PLoS ONE. 2024;19:e0311510. 39446771 10.1371/journal.pone.0311510 PMC11500892

[96] Jin Q, et al. Matching patients to clinical trials with large Language models. Nat Commun. 2024;15:9074. 39557832 10.1038/s41467-024-53081-z PMC11574183

[97] Hutson M. How AI is being used to accelerate clinical trials. Nature. 2024;627:S2–5. 38480968 10.1038/d41586-024-00753-x

[98] Commissioner O. of the. Project Optimus. *FDA* (2024).

[99] Senthil Kumar K, et al. Artificial intelligence in clinical oncology: from data to digital pathology and treatment. Am Soc Clin Oncol Educ Book. 2023;e390084. 10.1200/EDBK_390084. 10.1200/EDBK_390084 37235822

[100] Fountzilas E, Pearce T, Baysal MA, Chakraborty A, Tsimberidou AM. Convergence of evolving artificial intelligence and machine learning techniques in precision oncology. Npj Digit Med. 2025;8:1–19. 39890986 10.1038/s41746-025-01471-y PMC11785769

[101] Giuffrè M, Shung DL. Harnessing the power of synthetic data in healthcare: innovation, application, and privacy. Npj Digit Med. 2023;6:1–8. 37813960 10.1038/s41746-023-00927-3 PMC10562365

[102] Goncalves A, et al. Generation and evaluation of synthetic patient data. BMC Med Res Methodol. 2020;20:108. 32381039 10.1186/s12874-020-00977-1 PMC7204018

[103] Chen RJ, Lu MY, Chen TY, Williamson DFK, Mahmood F. Synthetic data in machine learning for medicine and healthcare. Nat Biomed Eng. 2021;5:493–7. 34131324 10.1038/s41551-021-00751-8 PMC9353344

[104] Marouf M, et al. Realistic in Silico generation and augmentation of single-cell RNA-seq data using generative adversarial networks. Nat Commun. 2020;11:166. 31919373 10.1038/s41467-019-14018-z PMC6952370

[105] Tran NH, et al. Deep learning enables de Novo peptide sequencing from data-independent-acquisition mass spectrometry. Nat Methods. 2019;16:63–6. 30573815 10.1038/s41592-018-0260-3

[106] Tran NH, Zhang X, Xin L, Shan B, Li M. De novo peptide sequencing by deep learning. *Proceedings of the National Academy of Sciences* 114, 8247–8252 (2017). 10.1073/pnas.1705691114 PMC5547637 28720701

[107] Torng W, Altman RB. High precision protein functional site detection using 3D convolutional neural networks. Bioinformatics. 2019;35:1503–12. 31051039 10.1093/bioinformatics/bty813 PMC6499237

[108] Mirzayans R, Murray D. What are the reasons for continuing failures in Cancer therapy?? are misleading/inappropriate preclinical assays to be blamed?? Might some modern therapy?ies cause more harm than benefit? Int J Mol Sci. 2022;23:13217. 36362004 10.3390/ijms232113217 PMC9655591

